# Supplementary material for: Relative Abundance of Lipid Metabolites in Spermatozoa across Three Compartments
Source: Int J Mol Sci. 2022 Oct 1;23(19):11655. doi: 10.3390/ijms231911655 (PMC9569887; doi:10.3390/ijms231911655)

Supplementary Material: Tables and Table legends

Tables S1-3 – see supplementary Excel file.

Table S1: Fatty acids measured in this study.

Table S2: Lipid species measured in positive ionisation mode

Table S3: Lipid species measured in negative ionisation mode

Abbreviations used in tables S1-S3:

Ceramides (CERs), cholesterol (CHOL), cholesterol esters (CEs), diglycerides (DG), fatty acids (FAs), lysophosphatidylcholines (LPCs), lysophosphatidylethanolamines (LPEs), phosphatidylcholines (PCs), phosphatidylcholine-plasmalogens (PC-Os), phosphatidylethanolamines (PEs), phosphatidylethanolamine-plasmalogens (PE-Os), phosphatidylglycerides (PGs), phosphatidylinositols (PIs), phosphatidylserines (PSs), sphingomyelins (SMs), and triglycerides (TGs).

Supplementary Table S4. Characteristics of participants included in this study. Men were categorised into groups according to progressive motility (< or ≥ median; 50% progressive motility); sperm count (≤ or > median; 92 million/ ejaculate) and total semen volume (≤ or > median; 3.4 ml). Data are presented as median (range).

	**All participants**	**Motility analysis**		**Sperm number analysis**		**Semen volume analysis**	
		LOW MOTILITY	HIGH MOTILITY	LOW SPERM NUMBER	HIGH SPERM NUMBER	LOW SEMEN VOLUME	HIGH SEMEN VOLUME
	n=26	n=12	n=14	n=13	n=13	n=12	n=14
**Age, years**	35 (20 - 45)	36.5 (24 - 45)	32.5 (20 - 40)	32 (20 - 38)	37 (27 - 45)	35 (26 - 45)	34 (20 - 40)
**BMI, kg/m2**	30.4 (24 - 36.5)	30.2 (24 - 36.5)	30.4 (26 - 33)	26 (24 - 36.5)	33 (30.2 - 34.4)	26 (26 - 33)	33 (24 - 36.5)
**Abstinence, days**	4 (2 - 9)	4 (3 - 8)	3 (2 - 9)	3 (2 - 9)	4 (3 - 8)	3 (2 - 8)	4 (3 - 9)
**Semen volume, ml**	3.4 (1.8 - 8.3)	4.35 (2.8 - 8.3)	3.2 (1.8 - 5.7)	3.1 (2.2 - 5.3)	4.7 (1.8 - 8.3)	2.8 (1.8 - 3.2)	4.7 (3.4 - 8.3)
**Semen pH**	8.5 (8 - 8.5)	8.5 (8 - 8.5)	8.5 (8 - 8.5)	8.5 (8 - 8.5)	8.5 (8 - 8.5)	8.25 (8 - 8.5)	8.5 (8 - 8.5)
**Sperm concentration million/ml**	26.75 (2.6 - 209)	21.2 (2.6 - 209)	27.25 (7.5 - 196)	13.3 (2.6 - 37.9)	38.4 (18.6 - 209)	28.95 (3.1 - 209)	26.25 (2.6 - 196)
**Total motility %**	56 (28 - 73)	45 (28 - 56)	62.5 (54 - 73)	61 (36 - 73)	56 (28 - 65)	58.5 (40 - 73)	53 (28 - 73)
**Progressive motility %**	50 (24 - 68)	40 (24 - 49)	58 (50 - 68)	50 (32 - 68)	50 (24 - 63)	53.5 (35 - 64)	49 (24 - 68)
**Non-progressive motility %**	5 (1 - 13)	6 (2 - 9)	5 (1 - 13)	6 (3 - 13)	4 (1 - 8)	5.5 (2 - 10)	5 (1 - 13)
**Immotile %**	44 (27 - 72)	55 (44 - 72)	38 (27 - 46)	39 (27 - 64)	44 (35 - 72)	41.5 (28 - 60)	47 (27 - 72)
**Total sperm number millions/ejaculate**	91.825 (8.68 - 921.2)	89.9 (8.68 - 606.1)	93.325 (24 - 921.2)	49.21 (8.68 - 90.65)	175.78 (93 - 921.2)	79.35 (8.68 - 606.1)	130.03 (13.78 - 921.2)
**HbA1c mmol/mol**	37 (28 - 49)	37.5 (28 - 49)	37 (32 - 42)	37 (28 - 42)	38 (32 - 49)	36 (33 - 49)	37.5 (28 - 41)
**Random plasma glucose mmol/l**	5.2 (4.4 - 8.2)	5.3 (4.4 - 8.2)	5.2 (4.7 - 6.5)	5.2 (4.4 - 6)	5.4 (4.8 - 8.2)	5.55 (4.7 - 8.2)	5.2 (4.4 - 5.6)

NB: WHO reference criteria for normal semen characteristics are:

Progressive motility 32-75%

Total sperm count 39-928 million / ejaculate

Semen volume 1.5-7.6 ml

Legends for Supplementary Figures

Figure S1: Distribution of lipid species across the three compartments. Lipid were included as follows: ceramides (CERs), cholesterol (CHOL), cholesterol esters (CEs), diglycerides (DG), fatty acids (FAs), lysophosphatidylcholines (LPCs), lysophosphatidylethanolamines (LPEs), phosphatidylcholines (PCs), phosphatidylcholine-plasmalogens (PC-Os), phosphatidylethanolamines (PEs), phosphatidylethanolamine-plasmalogens (PE-Os), phosphatidylglycerides (PGs), phosphatidylinositols (PIs), phosphatidylserines (PSs), polyunsaturated fatty acids (PUFAs), sphingomyelins (SMs), and triglycerides (TGs). Species were only included if p<u><</u>0.001.

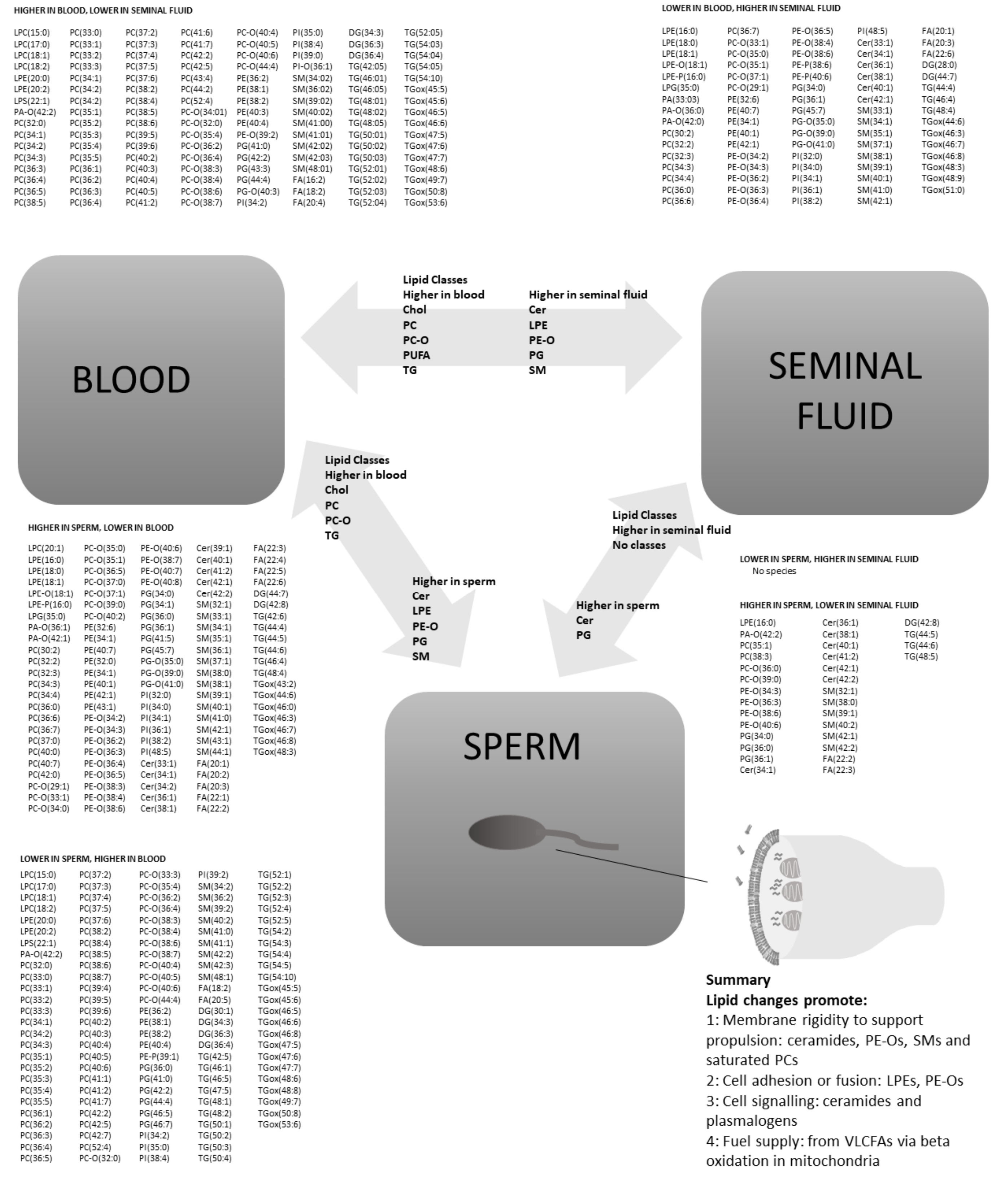

Figure S2: Distribution of lipids across three compartments shown in manhattan plots and heatmaps of relative abundance (a-c) and significance (d-f). Manhattan plots: lipid metabolites were tested for significance (-log P) and fold change between low motility and high motility groups within each compartment using unpaired t tests. Heatmap 1: Lipid metabolites were compared for relative abundance raw counts (a-b) and normalised to blood (c-d). Heatmap 2: Lipid metabolites were tested for significance (-log P) between low motility and high motility groups within each compartment using paired t tests. Lipid were identified as follows: ceramides (CERs), cholesterol (CHOL), cholesterol esters (CEs), diglycerides (DG), fatty acids (FAs), lysophosphatidylcholines (LPCs), lysophosphatidyl-ethanolamines (LPEs), phosphatidylcholines (PCs), phosphatidylcholine-plasmalogens (PC-Os), phosphatidyl-ethanolamines (PEs), phosphatidylethanolamine-plasmalogens (PE-Os), phosphatidylglycerides (PGs), phospha-tidylinositols (PIs), phosphatidylserines (PSs), polyunsaturated FAs (PUFAs), sphingomyelins (SMs), triglycerides (TGs).

MANHATTAN PLOTS

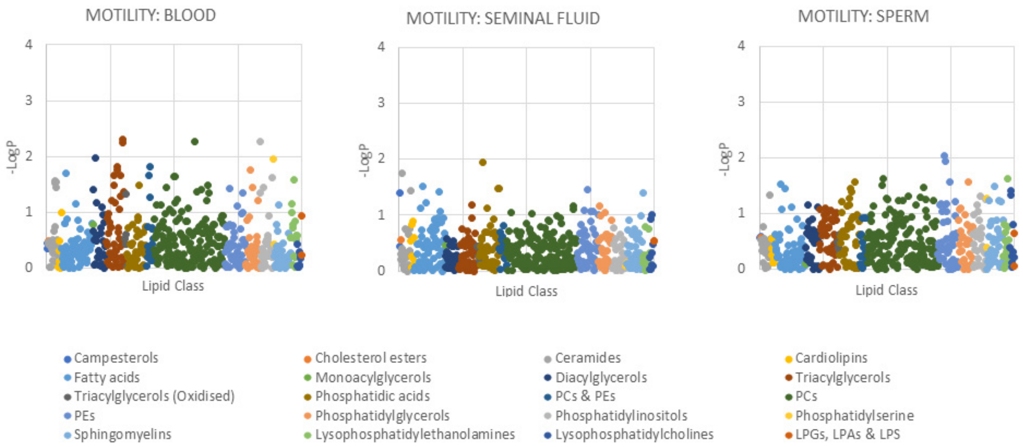

HEATMAP 1 – RELATIVE ABUNDANCE

a

	HIGH MOTILITY		
	BLOOD	SF	SPERM
CER	2.21	34.30	51.67
CER +	0.34	57.46	75.29
CFA	21.18	27.39	33.21
CHOL +	126.90	23.07	5.35
EFA	4.12	5.88	7.41
FA-OH	11.89	13.06	10.94
LPC	81.41	39.89	34.78
LPC +	175.34	177.60	188.01
LPE	157.24	375.24	360.36
LPE +	0.38	44.83	58.90
MFA	98.99	111.29	128.80
PC	323.56	73.87	79.60
PC +	393.27	74.74	80.62
PC-O	177.19	38.59	36.75
PE	106.29	99.59	90.51
PE +	4.08	58.20	44.07
PE-O	31.82	130.57	137.38
PG	5.71	12.57	17.09
PG +	4.25	3.44	3.34
PI	36.76	35.50	37.33
PS +	5.05	13.98	10.30
PUFA	69.71	44.06	49.71
SFA	818.32	830.71	809.49
SM	74.65	154.61	149.02
SM +	94.18	385.48	405.30
TG +	195.72	160.14	128.58

b

	LOW MOTILITY		
	BLOOD	SF	SPERM
CER	5.04	42.23	53.72
CER +	0.53	60.08	77.88
CFA	24.00	25.46	39.60
CHOL +	123.21	15.04	27.23
EFA	4.26	5.17	8.46
FA-OH	14.60	9.82	11.71
LPC	87.20	35.14	29.78
LPC +	161.97	155.44	140.70
LPE	162.49	362.55	320.31
LPE +	0.11	34.98	35.85
MFA	113.35	117.21	162.51
PC	316.99	67.02	74.23
PC +	343.95	65.01	63.88
PC-O	177.35	33.29	35.09
PE	102.95	107.27	95.17
PE +	3.97	80.00	59.82
PE-O	27.95	125.18	148.77
PG	5.47	13.62	21.44
PG +	3.38	10.04	2.71
PI	31.91	38.67	37.40
PS +	6.32	9.34	13.40
PUFA	86.02	44.57	60.44
SFA	784.76	827.53	764.28
SM	78.96	168.46	177.77
SM +	90.02	419.38	434.03
TG +	266.11	150.52	144.02

c

	HIGH MOTILITY		
	BLOOD	SF	SPERM
CER	1.00	15.48	23.33
CER +	1.00	169.40	221.98
CFA	1.00	1.29	1.57
CHOL +	1.00	0.18	0.04
EFA	1.00	1.43	1.80
FA-OH	1.00	1.10	0.92
LPC	1.00	0.49	0.43
LPC +	1.00	1.01	1.07
LPE	1.00	2.39	2.29
LPE +	1.00	118.40	155.59
MFA	1.00	1.12	1.30
PC	1.00	0.23	0.25
PC +	1.00	0.19	0.20
PC-O	1.00	0.22	0.21
PE	1.00	0.94	0.85
PE +	1.00	14.26	10.79
PE-O	1.00	4.10	4.32
PG	1.00	2.20	2.99
PG +	1.00	0.81	0.79
PI	1.00	0.97	1.02
PS +	1.00	2.77	2.04
PUFA	1.00	0.63	0.71
SFA	1.00	1.02	0.99
SM	1.00	2.07	2.00
SM +	1.00	4.09	4.30
TG +	1.00	0.82	0.66

d

	LOW MOTILITY		
	BLOOD	SF	SPERM
CER	1.00	8.38	10.66
CER +	1.00	114.31	148.18
CFA	1.00	1.06	1.65
CHOL +	1.00	0.12	0.22
EFA	1.00	1.22	1.99
FA-OH	1.00	0.67	0.80
LPC	1.00	0.40	0.34
LPC +	1.00	0.96	0.87
LPE	1.00	2.23	1.97
LPE +	1.00	307.26	314.84
MFA	1.00	1.03	1.43
PC	1.00	0.21	0.23
PC +	1.00	0.19	0.19
PC-O	1.00	0.19	0.20
PE	1.00	1.04	0.92
PE +	1.00	20.16	15.07
PE-O	1.00	4.48	5.32
PG	1.00	2.49	3.92
PG +	1.00	2.97	0.80
PI	1.00	1.21	1.17
PS +	1.00	1.48	2.12
PUFA	1.00	0.52	0.70
SFA	1.00	1.05	0.97
SM	1.00	2.13	2.25
SM +	1.00	4.66	4.82
TG +	1.00	0.57	0.54

Key

Key
Low Abundance
Medium Abundance
High Abundance

HEATMAP 2 - SIGNIFICANCE

a

HIGH MOTILITY GROUP

		-LogP		-LogP		-LogP	
CE+	Blood	2.35	SF	0.45	Sperm	2.86	Blood
CER+	Blood	4.11	SF	2.28	Sperm	8.20	Blood
CHOL+	Blood	3.57	SF	0.64	Sperm	5.63	Blood
EFA	Blood	1.15	SF	2.85	Sperm	2.77	Blood
FA-OH	Blood	0.23	SF	4.62	Sperm	4.95	Blood
LCFA	Blood	1.21	SF	3.63	Sperm	6.27	Blood
LPC	Blood	4.36	SF	7.82	Sperm	8.42	Blood
LPC+	Blood	0.18	SF	0.43	Sperm	0.87	Blood
LPE	Blood	7.77	SF	6.31	Sperm	0.06	Blood
LPE+	Blood	2.49	SF	1.10	Sperm	4.01	Blood
LPI+	Blood	0.80	SF	0.72	Sperm	0.82	Blood
MFA	Blood	0.57	SF	4.48	Sperm	4.78	Blood
PC	Blood	5.41	SF	3.31	Sperm	8.66	Blood
PC+	Blood	5.17	SF	0.59	Sperm	6.33	Blood
PC-O	Blood	11.60	SF	5.77	Sperm	9.89	Blood
PE	Blood	2.96	SF	2.03	Sperm	2.01	Blood
PE+	Blood	3.45	SF	0.80	Sperm	3.69	Blood
PE-O	Blood	1.01	SF	6.65	Sperm	8.28	Blood
PG	Blood	1.58	SF	7.11	Sperm	0.39	Blood
PG+	Blood	0.19	SF	0.04	Sperm	0.34	Blood
PI	Blood	7.51	SF	8.95	Sperm	3.31	Blood
PI-O	Blood	8.63	SF	8.97	Sperm	7.49	Blood
PS+	Blood	1.34	SF	1.74	Sperm	0.83	Blood
PUFA	Blood	0.87	SF	0.92	Sperm	2.84	Blood
SFA	Blood	0.09	SF	8.88	Sperm	11.47	Blood
SM	Blood	9.57	SF	0.00	Sperm	0.00	Blood
SM+	Blood	6.05	SF	1.54	Sperm	8.73	Blood
TG+	Blood	0.90	SF	1.56	Sperm	1.39	Blood

b

LOW MOTILITY GROUP

		-LogP		-LogP		-LogP	
CE+	Blood	1.38	SF	0.00	Sperm	1.38	Blood
CER+	Blood	4.52	SF	1.36	Sperm	7.76	Blood
CHOL+	Blood	3.03	SF	0.71	Sperm	2.14	Blood
EFA	Blood	1.66	SF	2.84	Sperm	2.29	Blood
FA-OH	Blood	0.71	SF	3.83	Sperm	3.57	Blood
LCFA	Blood	1.46	SF	2.88	Sperm	7.70	Blood
LPC	Blood	5.35	SF	8.33	Sperm	8.42	Blood
LPC+	Blood	0.10	SF	0.80	Sperm	0.50	Blood
LPE	Blood	6.78	SF	7.29	Sperm	1.03	Blood
LPE+	Blood	3.19	SF	0.06	Sperm	2.95	Blood
LPI+	Blood	0.92	SF	0.69	Sperm	1.14	Blood
MFA	Blood	0.88	SF	3.91	Sperm	4.69	Blood
PC	Blood	4.72	SF	5.32	Sperm	5.51	Blood
PC+	Blood	5.93	SF	0.08	Sperm	6.06	Blood
PC-O	Blood	9.44	SF	5.99	Sperm	8.75	Blood
PE	Blood	4.18	SF	3.01	Sperm	1.90	Blood
PE+	Blood	4.01	SF	0.60	Sperm	2.94	Blood
PE-O	Blood	0.79	SF	5.58	Sperm	8.06	Blood
PG	Blood	2.19	SF	4.94	Sperm	2.65	Blood
PG+	Blood	1.48	SF	1.31	Sperm	0.28	Blood
PI	Blood	6.40	SF	7.47	Sperm	4.80	Blood
PI-O	Blood	8.18	SF	9.05	Sperm	7.81	Blood
PS+	Blood	0.41	SF	0.41	Sperm	0.82	Blood
PUFA	Blood	1.10	SF	2.82	Sperm	3.42	Blood
SFA	Blood	0.20	SF	8.18	Sperm	10.58	Blood
SM	Blood	8.42	SF	0.00	Sperm	0.00	Blood
SM+	Blood	6.62	SF	0.33	Sperm	7.91	Blood
TG+	Blood	3.31	SF	0.15	Sperm	3.34	Blood

Key
0.00
2.00
4.00
6.00
8.00
10.00
12.00

Figure S3: Men with lower motility often have other conditions affecting fertility, including low spermatozoa count and low semen volume (categorised below median for each).

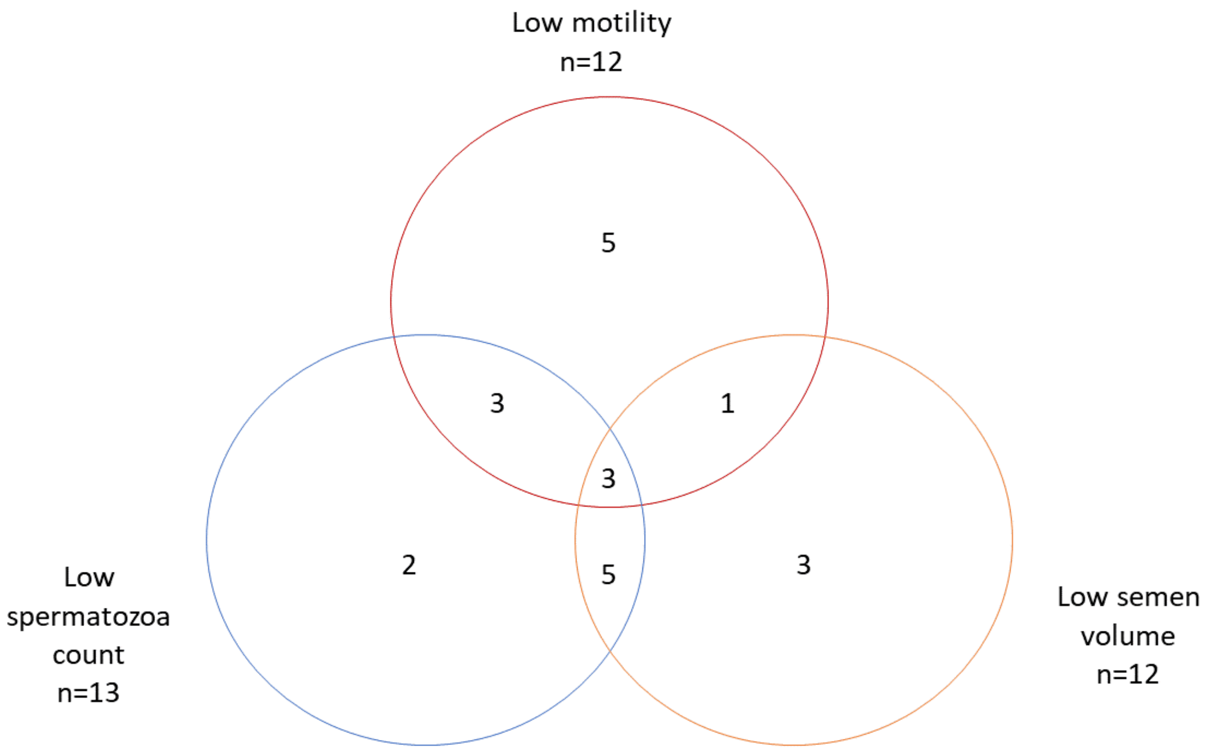

Figure S4: Significant differences in individual lipid species in association with sperm number between compartments. Lipid were included as follows: ceramides (CERs), cholesterol (CHOL), cholesterol esters (CEs), diglycerides (DG), fatty acids (FAs), lysophosphatidylcholines (LPCs), lysophosphatidylethanolamines (LPEs), phosphatidylcholines (PCs), phosphatidylcholine-plasmalogens (PC-Os), phosphatidylethanolamines (PEs), phosphatidylethanolamine-plasmalogens (PE-Os), phosphatidylglycerides (PGs), phosphatidylinositols (PIs), phosphatidylserines (PSs), polyunsaturated fatty acids (PUFAs), sphingomyelins (SMs), and triglycerides (TGs). Species were only included if $p \leq 0.001$.

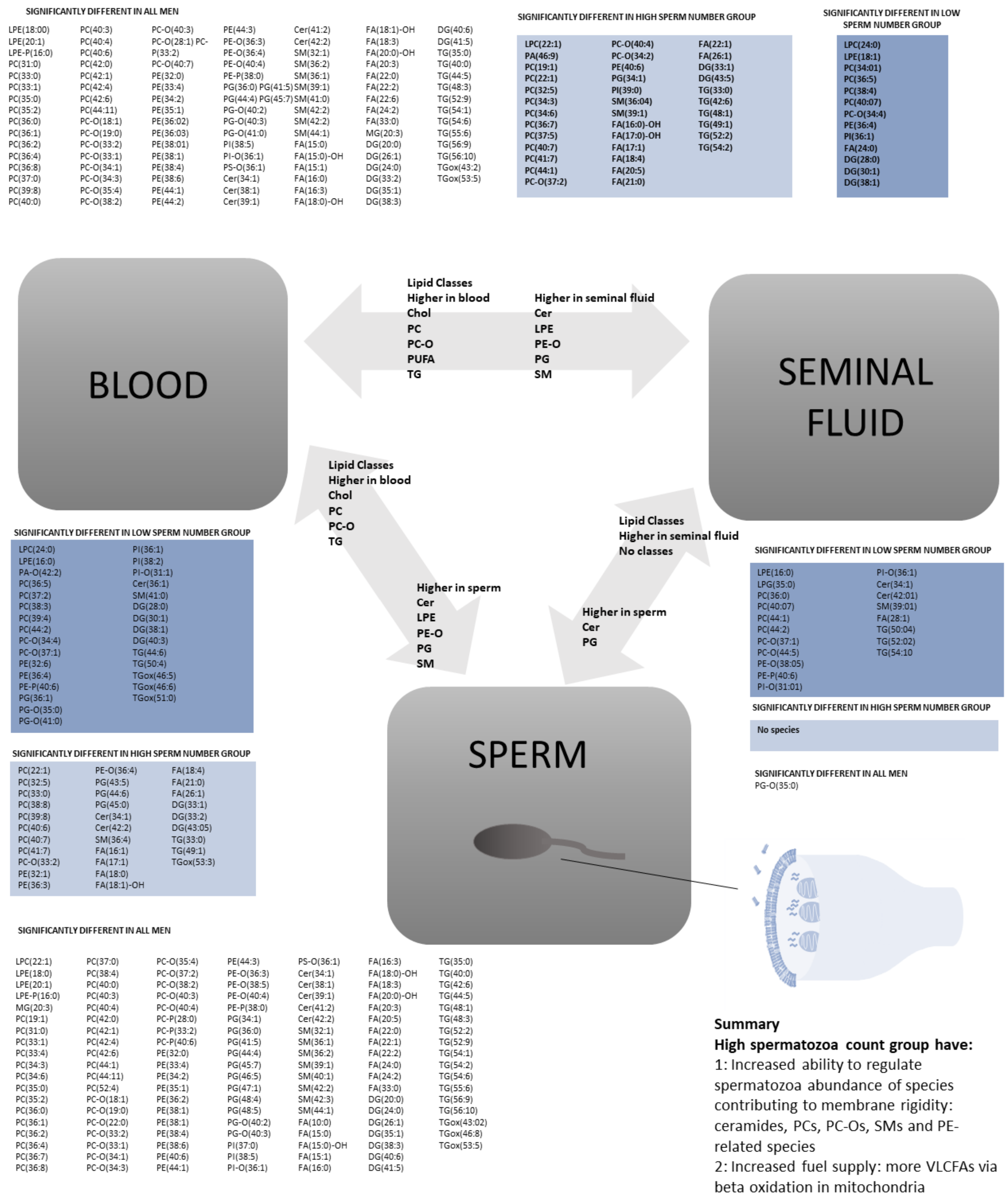

Figure S5: Significant differences in individual lipid species in association with semen volume between compartments. Lipid were included as follows: ceramides (CERs), cholesterol (CHOL), cholesterol esters (CEs), diglycerides (DG), fatty acids (FAs), lysophosphatidylcholines (LPCs), lysophosphatidylethanolamines (LPEs), phosphatidylcholines (PCs), phosphatidylcholine-plasmalogens (PC-Os), phosphatidylethanolamines (PEs), phosphatidylethanolamine-plasmalogens (PE-Os), phosphatidylglycerides (PGs), phosphatidylinositols (PIs), phosphatidylserines (PSs), polyunsaturated fatty acids (PUFAs), sphingomyelins (SMs), and triglycerides (TGs). Species were only included if $p \leq 0.001$.

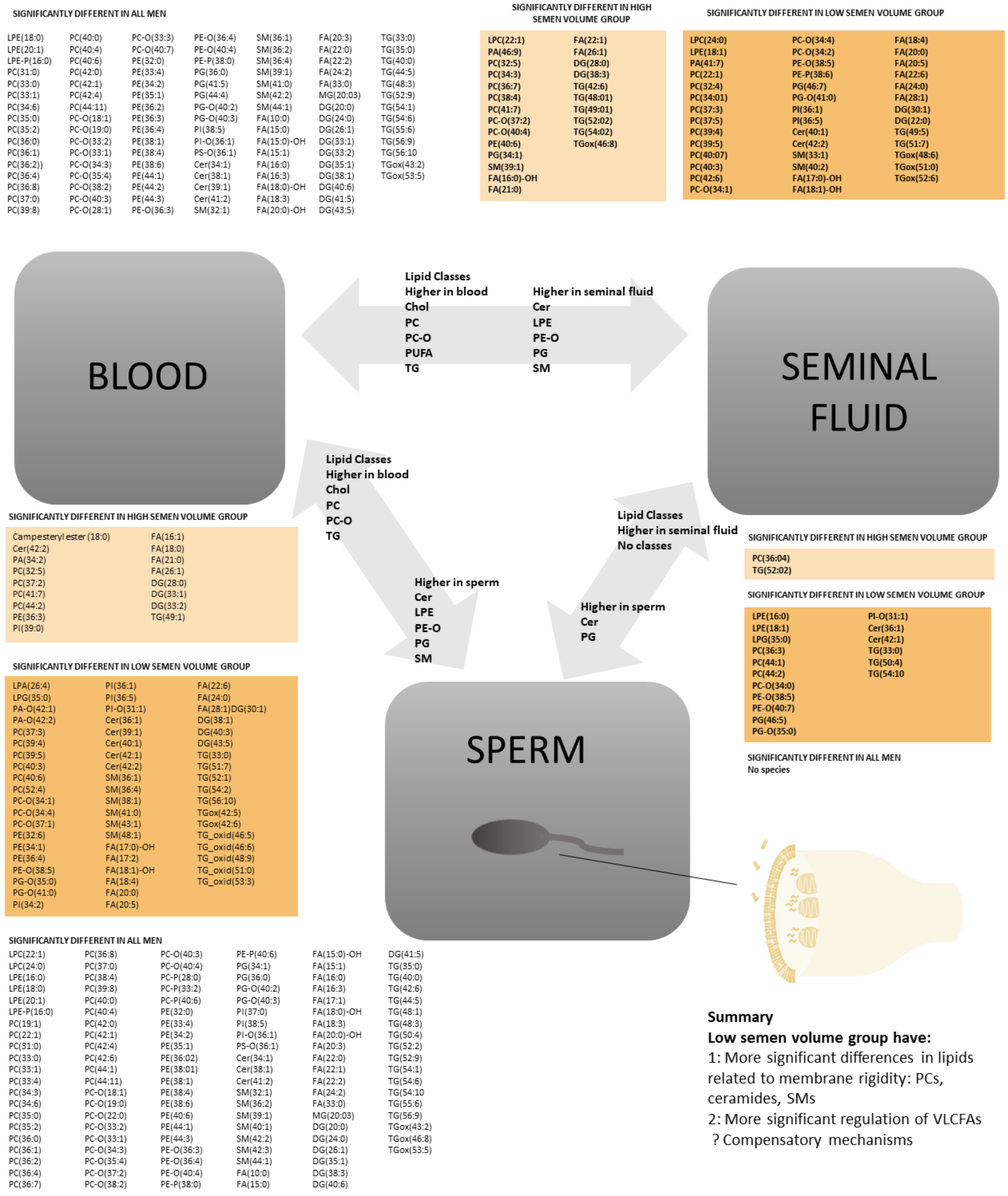

Supplement: Supplementary file 1 [file ijms-23-11655-s001.zip › ijms-1909048-supplementary.pdf]
